# Escherichia coli Cellulitis in a Patient With Waldenström Macroglobulinaemia: A Case Report

**DOI:** 10.7759/cureus.86917

**Published:** 2025-06-28

**Authors:** Murtada Ibrahim, Sidharth Ranjit

**Affiliations:** 1 Geriatric Medicine, Leicester Royal Infirmary Hospital, Leicester, GBR; 2 Geriatric Medicine, University Hospitals of Leicester NHS Trust, Leicester, GBR

**Keywords:** escherichia coli cellulitis, gram-negative bacteraemia, haematological malignancies, immunocompromised, waldenström macroglobulinaemia

## Abstract

*Escherichia coli *cellulitis is rarely reported, typically affecting immunocompromised individuals. The management of such cases presents significant challenges, as these patients often exhibit resistance to the empirical antibiotics commonly used for cellulitis. Furthermore, identifying *E. coli* in blood cultures necessitates additional investigations to rule out alternative sources of infection, such as biliary, urinary, or deep-seated collections. We present the case of a 79-year-old woman with Waldenström macroglobulinaemia, AL amyloidosis, and secondary immunosuppression who developed *E. coli* cellulitis. The diagnosis was confirmed via positive blood cultures, and no alternative sources of *E. coli* bacteraemia were identified. She was successfully treated with co-amoxiclav and flucloxacillin. This case underscores the importance of considering uncommon pathogens in immunocompromised patients and tailoring antimicrobial therapy based on microbiological sensitivity.

## Introduction

Cellulitis is a bacterial infection that spreads through the dermis and subcutaneous tissue, presenting with localized inflammatory signs such as erythema, pain, swelling, and warmth, along with systemic symptoms like fever, myalgia, and elevated inflammatory markers. 

While most uncomplicated skin and soft tissue infections (uSSTIs) are caused by *Staphylococcus aureus* and streptococci, immunocompromised individuals may develop infections from less common organisms, including Gram-negative bacteria, anaerobes, mycobacteria, and fungi. Drug users may contract cellulitis from unusual species such as *Enterococcus* and *Clostridium*, while surgical patients with abdominal or perineal infections frequently have Gram-negative or anaerobic bacterial causes. Specific environmental exposures, such as water contact or animal bites, can also introduce rare pathogens. Severe infections, including necrotizing fasciitis, are predominantly caused by *S. aureus* and group A beta-haemolytic streptococci, whereas diabetic foot infections commonly involve Gram-positive cocci, with chronic cases potentially including Gram-negative or anaerobic pathogens depending on prior antibiotic exposure​ [1]​. 

## Case presentation

A 79-year-old female presented to the hospital with fever, rigours, and vomiting. She had previously been started on oral flucloxacillin by her general practitioner for right leg cellulitis. Her past medical history was significant for Waldenstrom macroglobulinaemia, AL amyloidosis, secondary immunosuppression following chemotherapy, hypertension, chronic kidney disease (CKD), and asthma. Her regular medications included acyclovir and co-trimoxazole for long-term prophylaxis. She was usually independent with activities of daily living and had a clinical frailty score of 3.

On clinical examination, her right leg was swollen, hot, erythematous, and tender. Laboratory investigations showed a C-reactive protein (CRP) of 383 mg/L and an initial white cell count (WCC) of 2.5 × 10⁹/L, which subsequently increased to 8.6 × 10⁹/L but remained within the normal range throughout her hospital stay. Her haemoglobin was lower than usual, dropping to 71 g/L from a baseline of 93 g/L, with slight improvement during her admission. Renal function showed a mild decline with an estimated glomerular filtration rate (eGFR) of 28 mL/min/1.73 m² from a baseline of 34 mL/min/1.73 m² (Table 1).

**Table 1:** Blood results during the course of treament The table shows a good response to antibiotic therapy with a reduction of the C-reactive protein (CRP). The estimated glomerular filtration rate (eGFR) was low during admission, improved with treatment; the patient's usual level is between 40 and 50 ml/min/1.73 m^2^*.* WCC: white blood cell count, Hb: haemoglobin, eGFR: estimated glomerular filtration rate, CRP: C-reactive protein

Investigations	Reference range	Day 1	Day 3	Day 4	Day 6	Day 8	Day 11
WCC (× 10⁹/L)	4-11	2.7	8.6	6.9	4.1	4.3	4.0
Hb (g/L)	115-165	93	71	82	81	84	84
eGFR (mL/min/1.73m²)	N/A	28	31	37	48	46	41
CRP (mg/L)	0-10	52	383	354	148	75	25

Blood cultures grew *Escherichia coli*, which was resistant to ciprofloxacin, co-trimoxazole, and trimethoprim, but sensitive to amoxicillin, co-amoxiclav, gentamicin, and piperacillin-tazobactam.

**Figure 1:**
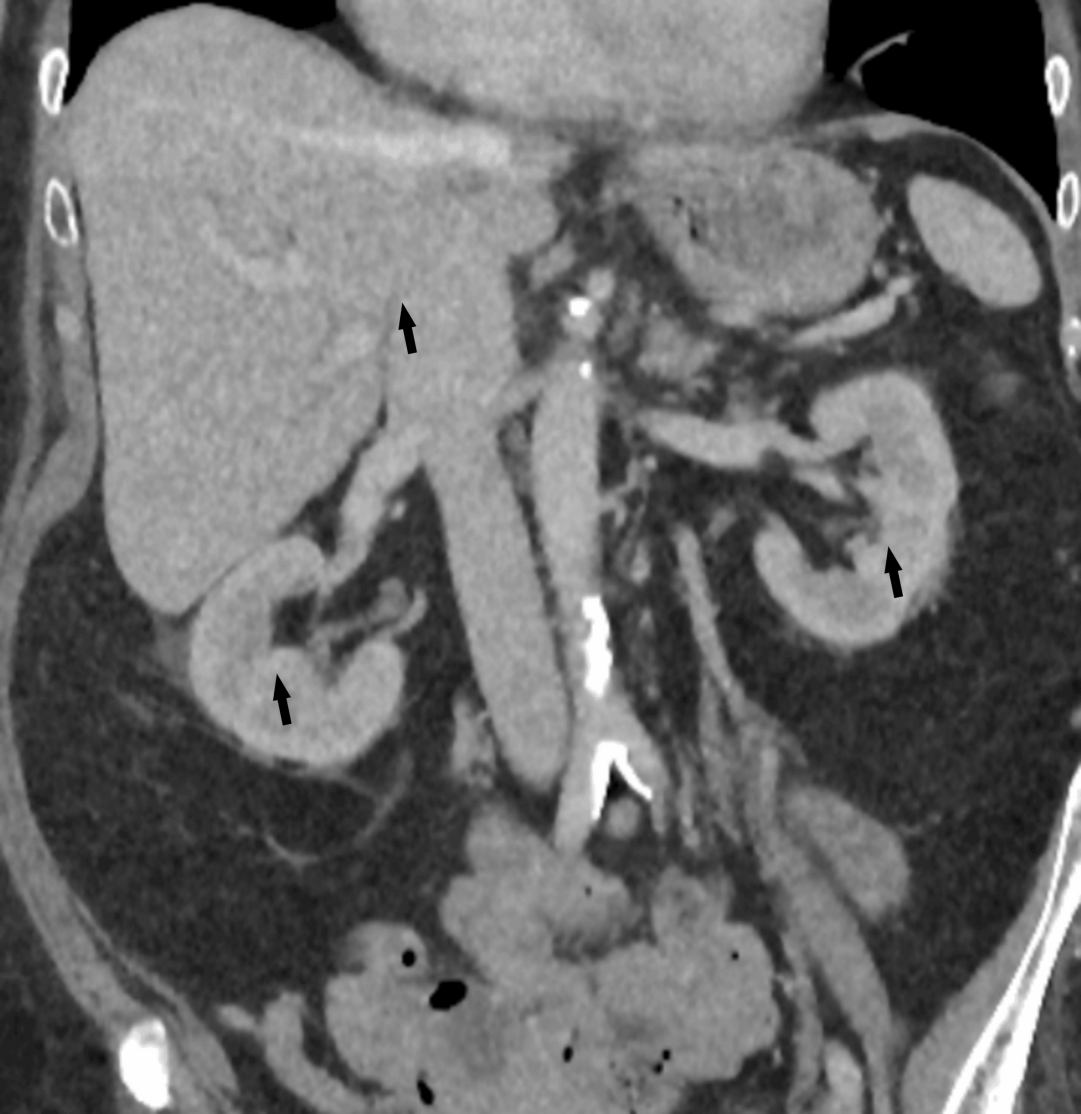
CT abdomen and pelvis with contrast CT Abdomen and pelvis with contrast showed no deep collection, no significant peri-nephric stranding, and no biliary infection. See black arrows.

Given the unusual finding of *E. coli* in blood cultures, further investigations were performed to identify a potential primary source. A computed tomography (CT) scan of the abdomen and pelvis did not identify any abdominal source (Figure 1). Doppler ultrasound of the right leg excluded deep vein thrombosis. Urine microscopy and culture were negative with no growth. Due to pain in both feet, plain radiographs were obtained, which showed no fractures or features of osteomyelitis.

Microbiology advice was requested, commenced on intravenous co-amoxiclav 1.2 g three times per day. She completed a seven-day course with significant improvement in her inflammatory markers. However, the swelling and pain over the right leg showed limited improvement; however, there was no further temperature spikes beyond the third day of co-amoxiclav therapy. Due to persistent symptoms, the case was discussed with the microbiology team, who recommended an additional five-day course of oral flucloxacillin, so a five-day course of oral flucloxacillin 1 gram four times per day was started. This combination approach resulted in a good clinical response (Table 1). She was discharged home to complete five days of oral flucloxacillin.

## Discussion

*E. coli* cellulitis remains a rare phenomenon, primarily affecting immunocompromised individuals. Few reported cases exist: Nguyen et al. (2023) reported an 84-year-old woman with breast cancer who developed *E. coli* cellulitis [2]​. Maciej et al. (2024) noted severe erysipelas in a 57-year-old kidney transplant recipient ​[3]​. Alcorta et al. (2021) reported *E. coli* bacteraemia and cellulitis in an 11-year-old child with nephrotic syndrome ​[4]​. Sunder et al. (2012) studied two patients with haematological malignancies who presented with life-threatening *E. coli* cellulitis [5]​. 

*E. coli*, a gram-negative bacterium from the Enterobacteriaceae family, is a leading cause of extraintestinal infections, including bacteremia and sepsis. It has surpassed *Staphylococcus aureus* and *Streptococcus pneumoniae* as the most common pathogen responsible for bloodstream infections in high-income countries. The rise of multidrug-resistant* E. coli* strains poses a growing clinical challenge, increasing the risk of severe bacteremia and higher mortality rates ​[6]​. 

Although *E. coli* rarely causes skin and soft tissue infections, immunocompromised patients may develop cellulitis from atypical pathogens, including Gram-negative bacteria [1]​. The few reported cases of *E. coli* cellulitis have been described in immunocompromised patients with liver cirrhosis, chronic renal failure, post-organ transplantation and with haematological malignancies ​[2]​ 

The emergence of quinolone-resistant *E. coli *in neutropenic cancer patients has been documented since the mid-1990s, with recent studies indicating rising prevalence ​[7]​.

In this case, flucloxacillin - typically effective against cellulitis - failed to resolve symptoms, suggesting an atypical causative organism. The identification of *E. coli* via blood cultures confirmed this suspicion, and no alternative sources of infection were found. This highlights the importance of considering uncommon pathogens in immunocompromised patients, particularly when empirical antibiotics yield poor clinical response.

## Conclusions

This case highlights a rare instance of *E. coli* cellulitis in elderly, immunocompromised patients with Waldenström macroglobulinaemia and AL amyloidosis. It underscores the necessity of considering atypical and Gram-negative organisms when patients exhibit poor clinical response to standard empirical therapy for cellulitis. Prompt pathogen identification through blood cultures and exclusion of alternative infection sources are crucial in guiding appropriate antimicrobial treatment. Clinicians should remain vigilant for unusual pathogens in high-risk populations and tailor therapy based on microbiological sensitivity to ensure optimal outcomes. 
